# Lessons learned from child sexual abuse research: prevalence, outcomes, and preventive strategies

**DOI:** 10.1186/1753-2000-7-22

**Published:** 2013-07-18

**Authors:** Delphine Collin-Vézina, Isabelle Daigneault, Martine Hébert

**Affiliations:** 1 School of Social Work, McGill University, 3506 University Street, room 321A, Montreal (QC), Canada H3A 2A7; 2 Psychology Department, Université de Montréal, P.O. Box 6128, Downtown Station, Montréal, QC, Canada H3C 3J7; 3 Sexology Department, Université du Québec à Montréal, P.O. Box 8888, Downtown Station, Montréal, QC, Canada H3C 3P8

**Keywords:** Child sexual abuse, Review, Prevalence, Mental health outcomes, Prevention

## Abstract

Although child sexual abuse (CSA) is recognized as a serious violation of human well-being and of the law, no community has yet developed mechanisms that ensure that none of their youth will be sexually abused. CSA is, sadly, an international problem of great magnitude that can affect children of all ages, sexes, races, ethnicities, and socioeconomic classes. Upon invitation, this current publication aims at providing a brief overview of a few lessons we have learned from CSA scholarly research as to heighten awareness of mental health professionals on this utmost important and widespread social problem. This overview will focus on the prevalence of CSA, the associated mental health outcomes, and the preventive strategies to prevent CSA from happening in the first place.

## 

Although only recently acknowledged as a concerning social problem, child sexual abuse (CSA) is, in our day, at the forefront of worldwide social policies and practices. Four decades of research has certainly contributed to better our knowledge on the experiences of victims of CSA. With more than 20,000 research papers on CSA listed under the most renowned research databases, child and adolescent mental health practitioners, researchers and decision-makers may find it challenging to keep up with this rapidly increasing literature. In response to this need, the aim of the current paper is to provide a brief overview on CSA to heighten awareness of practitioners on this utmost important and widespread social problem. The content of this paper was first presented at the annual symposium of the *Centre for Child Protection*, headed by the Institute of Psychology at the Pontifical Gregorian University and scholars of the University of Ulm, to a group of religious leaders responding to the sexual abuse of minors around the world, including Argentina, Ecuador, Germany, Ghana, India, Indonesia, Italy and Kenya. Upon invitation, this current publication is a unique opportunity to highlight a few of the main lessons we have learned from the scholarly literature on CSA, with a focus on its prevalence, mental health outcomes and preventive strategies.

## Magnitude: how prevalent is CSA?

Until recently, there was much disagreement as to what should be included in the definition of CSA [1]. In some definitions, only contact abuse was included, such as penetration, fondling, kissing, and touching [2]. Non-contact sexual abuse, such as exhibitionism and voyeurism, were not always considered abusive. Nowadays, the field is evolving towards a more inclusive understanding of CSA that is broadly defined as any sexual activity perpetrated against a minor by threat, force, intimidation, or manipulation. The array of sexual activities thus includes fondling, inviting a child to touch or be touched sexually, intercourse, rape, incest, sodomy, exhibitionism, involving a child in prostitution or pornography, or online child luring by cyberpredators [3,4]. CSA experiences vary greatly over multiple dimensions including, but not limited to: duration, frequency, intrusiveness of acts perpetrated, and relationship with perpetrator. Although sexual activity between children has long been thought to be harmless, child on child CSA experiences, such as those involving siblings, is increasingly being recognized as detrimental for the emotional well-being of children as adult on child CSA [5-7]. While adult-to-child interactions in which the purpose is sexual gratification are considered abusive, sexual behaviours between children are less clear-cut as there is no universal definition of sexual abuse that differentiates it from normal sex play and exploration [8]. Although a 2 to 5-year age difference between children was first suggested as necessary to consider sexual behaviours between siblings to be incest [9], this criterion is being questioned as studies have shown this age difference to be much lower in many substantiated cases of child-to-child abuse [10]. This formulation of CSA is in keeping with the recommendations from the 1999 World Health Organization Consultation on Child Abuse Prevention, where CSA is defined as any activity of a sexual nature ‘between a child and an adult or another child who by age or development is in a relationship of responsibility, trust or power, the activity being intended to gratify or satisfy the needs of the other person’. That said, some definitional issues have not yet been resolved in the field. First, much disparity exists regarding age for sexual consent, or age for sexual maturity, which has an influence on the extent to which statutory sex offenses are considered CSA. Sexual activities that involve a person below a statutorily designated age fall under the large umbrella of CSA; however, the age of consent varies greatly across countries, from as young as 12 or 13 (e.g. Tonga, Spain) to 17 or 18 years of age (e.g. some states in the US, Australia). In virtually all European jurisdictions, sexual relations are legal from age 16 onwards, but some countries have set the age for sexual consent at 14 or 15 [11]. In other words, when no coercion or force is used, cases that involve sexual activities between an adult and, for example, a 14-year-old teenager, will be either perceived as a consensual sexual relationship or criminalized and defined as sexual abuse, depending on the legal statutorily designated age of the country where the event occurred. In Canada, a bill was recently adopted to change the age of consent from 14 to 16, a premiere in Canada’s history, which emphasizes the impact governmental decisions can have on definitional issues of CSA in societies over time [12]. Second, although coerced sexual activities that occur in dating or romantic relationships is recognized as a form of sexual violence by the World Health Association (see for example a WHO multi-country study from Garcia-Moreno and colleagues [13]), the extent to which this form of interpersonal violence is socially recognized and acknowledged in different legislations around the world is unclear.

In that vein, the exact extent of the problem of CSA is difficult to approximate given the lack of consensus on the definition used in research inquiries, as well as the differences in the data collection systems across areas [14]. For example, in their review of the current rates of CSA across 55 studies from 24 countries, Barth and colleagues [15] found much heterogeneity in studies they reviewed and concluded that rates of CSA for females ranged from 8 to 31% and from 3 to 17% for males. Though, despite these methodological challenges, recent systematic reviews and meta-analyses that included studies conducted worldwide across hundreds of different age-cohort samples have consistently shown an alarming rate of CSA, with averages of 18-20% for females and of 8-10% for males [16], with the lowest rates for both girls (11.3%) and boys (4.1%) found in Asia, and highest rates found for girls in Australia (21.5%) and for boys in Africa (19.3%) [17]. Research findings do, however, clearly demonstrate a major lack of congruence between the low number of official reports of CSA to authorities, and the high rates of CSA that youth and adults self-report retrospectively. Indeed, the recent comprehensive meta-analysis conducted by Stoltenborgh and colleagues [17] that combined estimations of CSA in 217 studies published between 1980 and 2008, showed the rates of CSA to be more than 30 times greater in studies relying on self-reports (127 by 1000) than in official-report inquiries, such as those based on data from child protection services and the police (4/1000). In other words, while 1 out of 8 people report having experienced CSA, official incidence estimates center around only 1 per 250 children.

This discrepancy can be explained by the different steps that CSA cases go through before they are substantiated, and thus counted in official-report inquiries. First, victims of CSA or their confidants have to disclose their suspicions to the authorities. Many reports of child abuse are never passed on. In fact, the majority of studies highlight the fact that many victims continue to be unrecognized [3]. A review of CSA studies by Finkelhor [2] found that across all studies, only about half of victims had disclosed the abuse to anyone. This problem is often referred to as the phenomenon of the “tip of the iceberg” [18], where only a fraction of CSA situations are visible and a much higher proportion remain undetected. Disclosure is a delicate and sensitive process that is influenced by several factors, including implicit or explicit pressure for secrecy, feelings of responsibility or blame, feelings of shame or embarrassment, or fear of negative consequences [2,19,20]. Ethnic and religious cultures may also influence the way by which the process of disclosure is experienced and can act as either facilitators or barriers to the telling and reporting of CSA [21], which may explain variations of CSA rates across geographical areas [17]. Moreover, mandatory reporting regulations that have been adopted over the past decades in several countries, which imply that professionals are obliged to bring their suspicions of CSA to the attention of the authorities, can also impact the official counts of CSA in different countries [22]. In jurisdictions that have chosen not to enact mandatory reporting, including New Zealand, the United Kingdom, and Germany, a large discrepancy between adult self-reports of CSA and official data is to be expected as more cases may not be divulged to the authorities than in countries where reporting is mandatory. Second, based upon the initial disclosure or reporting, cases are screened in or out for further investigation by child protection workers or the police. Not all sexual abuse cases are considered to fall under the jurisdiction of child protection services, such as those that were assessed to involve no imminent risk to the child with regards to his/her security and development. For instance, cases where the alleged perpetrator is not the child’s caregiver may be less likely to be retained for investigation as it may not be under child welfare responsibilities to investigate these cases [23]. Finally, in light of evidence gathered in the course of the investigation process, cases are deemed substantiated or not by child protection workers and the police. When the child’s testimony is deemed unreliable or when the proof is perceived as questionable, cases may be considered unfounded and will, as a result, not be counted towards official data. Indeed, there is some evidence that police are less likely to charge sexual offenses than any other type of violent crime [24]. Other factors, such as the victim’s gender, may also influence substantiation decisions as demonstrated in a recent American study that showed, using the *National Survey of Child and Adolescent Well-Being,* that workers were less likely to substantiate cases involving male victims [25]. As improper interviewing techniques may hamper the capacity of victims to report accurately the abusive experience they were subjected to, promoting and sustaining best-practice interviewer techniques, notably among police officers, should be prioritized [26]. Considering the impact that all these different layers of influence have on cutting down the number of CSA cases that are known to and substantiated by the authorities, victims identified in official-report inquiries are therefore believed to represent only a small fraction of the true occurrence. For all these reasons, relying on official-reports to determine the magnitude of CSA is a method that carries a constant error of underestimation. In other words, children that are identified are only those that were able to disclose, were believed, reported to, and followed up by proper authorities, and those cases that presented enough evidence to be substantiated as CSA.

In terms of risk factors, being female is considered a major risk factor for CSA as girls are about two times more likely to be victims than males [16,17]. Several authors do, however, point out that there is a strong likelihood that boys are more frequently abused than the ratio of reported cases would suggest given their probable reluctance to report the abuse [27]. A recent Canadian population-based study confirmed this assumption by showing that among CSA survivors, 16% of female victims had never disclosed the abuse, whereas this proportion rose to 30% for male victims [28]. With respect to age, children who are most vulnerable to CSA are in the school-aged and adolescent stages of development, though about a quarter of CSA survivors report they were first abused before the age of 6 [3]. In addition, girls are considered to be at high risk for CSA starting at an earlier age and lasting longer, while boys’ victimisation peaks later and for a briefer period of time. The presence of disability is also considered a risk factor for CSA and other forms of maltreatment as the impairments may heighten the vulnerability of the child [29]. Aside, the absence of one or both parents or the presence of a stepfather, parental conflicts, family adversity, substance abuse and social isolation have also been linked to a higher risk for CSA [30]. In terms of the presupposed impact of socioeconomic status and ethnic background, the existing literature has many weaknesses and obvious contradictions. Overall, while low family or neighborhood socioeconomic status is a great risk factor for physical abuse and neglect [31,32], its impact on CSA is not as proven. On one hand, CSA could appear to occur more frequently among underprivileged families because of the disproportionate number of CSA cases reported to child protective services that come from lower socioeconomic classes [3]. In that vein, some populations of children have been overrepresented in research that focuses on vulnerable populations, such as Black American children from low socioeconomic status families, which may create an erroneous belief that race and ethnicity are risk factors for CSA [33]. On the other hand, some recent population-based studies are showing that, amongst other factors, living in poverty is a predictive factor for children to be subjected to both physical and sexual abusive experiences [34,35].

## Mental health outcomes: what are the effects of CSA?

Several models have been developed in an attempt to explain the adverse negative impact of CSA [36]. Among the most established conceptual frameworks on the impact of CSA is the Four-Factor Traumagenics Model [37]. This model suggests that CSA alters a child’s cognitive and emotional orientation to the world and causes trauma by distorting their self-concept and affective capacities. This model underscores the issues of trust and intimacy that are particularly pronounced among victims of CSA. The unique nature of CSA as a form of maltreatment is highlighted by the four trauma-causing factors that victims may experience, which are traumatic sexualization, betrayal, powerlessness, and stigmatization. Traumatic sexualization refers to the sexuality of the victims that is shaped and distorted by the sexual abuse. Betrayal is the loss of trust in the perpetrator who shattered the relationship and in other adults who are perceived as not having protected the child from being abused in the first place, or having not supported her upon disclosure. Powerlessness is experienced through power issues at play in CSA, where victims are unable to alter the situation despite feeling the threat of harm and the violation of their personal space. Stigmatization is the incorporation of perceptions, reinforced by the perpetrator’s manipulative discourse or by dominant social negative attitudes towards victims, of being bad or deserving and responsible for the abuse.

Several reviews and meta-analyses published in the 90s and early years of 2000 suggested that a wide range of psychological and behavioral disturbances were associated with the experience of CSA, which led experts in the field to conclude that CSA was a substantial risk factor in the development of a host of negative consequences in both childhood, adolescence and adulthood [38-41]. More recently, systematic reviews have confirmed that, given the vast array of etiological factors that interact in predicting mental health outcomes, CSA is considered a significant, though general and nonspecific, risk factor for psychopathology in children and adolescents [42-44].

Among the wealth of psychopathologies that have been studied among CSA victims, post-traumatic stress and dissociation symptoms have received great attention. Overall, victims have been shown to present significantly more of these symptoms than non-abused children, or than victims of other forms of trauma. In one of our studies that compared 67 sexually abused school-aged girls with a matched group, CSA was found to significantly increase the odds of presenting with a clinical level of dissociation and PTSD symptoms, respectively, by eightfold and fourfold [45]. These results have echoed previous research conducted among cohorts of sexually abused school-aged children and teenagers where about a third to a half of all victims showed clinical levels of post-traumatic stress symptoms [46-50]. Only a few studies have been conducted with younger cohorts of children, yet high levels of dissociation were documented among sexually abused preschoolers [51,52]. In that vein, results from one of our recent inquiries revealed higher frequencies of dissociative symptoms among a group of 76 sexually abused children aged 4 to 6 than children of the comparison group [53]. These symptoms were found to persist over a period of a year following disclosure [54]. In contrast to children who have experienced other forms of trauma, it was also found that CSA victims are more likely to present post-traumatic stress symptoms [55]. Using a prospective method in which sexually abused children were followed over 36 months, Maikovich, Koenen, and Jaffe [25] demonstrated that boys were as likely as girls to exhibit post-traumatic stress symptoms.

Aside from post-traumatic stress and dissociation symptoms, a significant number of other mental health and behavioral disturbances have been linked to CSA. High levels of mood disorders, such as major depressive episodes, are found in cohorts of children and teenagers who have been sexually abused [56,57]. Sexually abused children are more likely than their non-abused counterparts to present behavior problems, such as inappropriate sexualized behaviors [58]. In the teenage years, they are found to more often exhibit conduct problems [59] and engage in at-risk sexual behaviors [60,61]. Victims are more prone to abusing substances, to engaging in self-harm behaviors, and to attempting or committing suicide [62-65]. Adolescents sexually abused in childhood are five times more likely to report non-clinical psychotic experiences such as delusions and hallucinations than their non-abused counterparts [66].

The mental health outcomes of CSA victims are likely to continue into adulthood as the link of CSA to lifetime psychopathology has been demonstrated [67-72]. Even more worrisome is the fact that CSA victims are more at risk than non-CSA youth to experience violence in their early romantic relationships [73,74] and that they are 2–5 times more at risk of being sexually revictimized in adulthood than women not sexually abused in childhood [75-77]. In adulthood, CSA survivors are more likely to experience difficulties in their psychosexual functioning [78,79]. A 23-year longitudinal study of the impact of intrafamilial sexual abuse on female development confirmed the deleterious impact of CSA across stages of life, including all of the mental health issues mentioned above, but also hypothalamic–pituitary–adrenal attenuation in victims, as well as asymmetrical stress responses, high rates of obesity, and healthcare utilization [80]. The impact of CSA as a predictor of major illnesses is garnering increasing attention, including gastrointestinal disorders, gynecologic or reproductive health problems, pain, cardiopulmonary symptoms, and diabetes [81-83]. In all cases, early assessment and intervention to offset the exacerbation and continuation of negative outcomes is highlighted, according to several studies [84], as symptoms can develop at a later age [3] or may not be apparent at first [85].

Indeed, despite overwhelming evidence of deleterious outcomes of CSA, it is commonly agreed that the impact of CSA is highly variable and that a significant portion of victims do not exhibit clinical levels of symptoms [86]. Some authors have suggested that about a third of victims may not manifest any clinical symptoms at the time the abuse is disclosed [87]. This can be explained, in part, by the extremely diverse characteristics of CSA which lead to a wide range of potential outcomes [86]. Other common reasons thought to account for asymptomatic survivors of sexual abuse include: (1) insufficient severity of abuse, (2) the fact that symptoms may not be detected by practitioners, (3) development of avoidant coping styles that mask victims’ distress, (4) or that asymptomatic survivors may be more resilient than the survivors who show symptoms [88]. Related to this latter explanation, among an array of variables potentially influencing the resilience capacities of CSA victims, children who receive support from their non-offending parents [89] and those who have not experienced prior abuse [90] seem to fare better in spite of the sexual abuse adversity. Among other personal and relational factors that promote resilience in victims are: less reliance on avoidant coping strategies to deal with the traumatic event [91-93], higher emotional self-control [94], interpersonal trust and feelings of empowerment [85], less personal attributions of blame and of stigmatization [95,96], and high family functioning and secure attachment relationships [97,98]. This scholarship points to the importance of using a broad ecological framework when researching and intervening on the factors that promote resilience in victims of CSA [88].

Three promising lines of research have recently emerged that shed new light on the relationships between CSA and psychopathology. First, results from the growing field of polyvictimization, which is the study of the impact of multiple types of victimization (from peers, family, crime, community violence, physical assaults, and sexual assaults), call for a de-compartmentalization of violence research by pointing out that cumulative experiences of victimizations are more detrimental to the child’s well-being than are any single experiences, including those of a sexual nature [99]. This suggests that measuring the impact of all forms of victimization alongside CSA is warranted in order to fully capture the influence of violence and abuse on the development of children and youth mental health outcomes. Second, recognizing the great diversity of symptom presentations in sexually abused cohorts, several scholars have attempted to identify the different profiles or sub-categories of victims. For example, Trickett and colleagues [100] found distinct profiles in their sample of girls sexually abused by family members, including victims of multiple perpetrators, characterized by significantly higher levels of dissociation, and victims of father-daughter incest who presented higher levels of disturbances across domains, including internalized (e.g. depression) and externalized (e.g. delinquency) behaviors. Hébert and colleagues [101] further contributed to this scholarship by identifying four different profiles among a sample of sexually abused children: (1) the chronically abused children displaying anxiety symptoms, (2) the severely abused children presenting a host of both internalized and externalized problems, (3) the less severely abused children displaying fewer symptoms, (4) and the less severely impaired children despite severe experiences of CSA, which the authors referred to as the resilient group. As a whole, these studies call for a better tailoring of the services offered to sexually abused children, so that services can well match the mental health needs of victims [102]. Third, drawing from epigenetics [103], cutting-edge inquiries are developing in CSA research on the interaction of CSA with other environmental factors and with genetic factors to predict mental health and behavioral outcomes, for example, violent behavior [104], or suicidal gesture [105]. These inquiries confirm the relevance of studying the psychobiology of child maltreatment [106] as a promising route to better our understanding of the unique contribution of CSA to mental health disturbances, relative to other factors, as well as of the complex nature of the interactions at play. This knowledge could eventually benefit the elaboration of effective intervention programs.

## Preventive strategies: how can we prevent CSA from happening in the first place?

In light of the high prevalence of CSA and the wealth of deleterious outcomes associated with this abusive experience, it stands to reason that research attention must turn toward preventing CSA. Two widespread forms of sexual assault prevention efforts have been extensively studied and disseminated, namely, offender “management” and educational programs delivered, for the most part, in school settings. Offender management is the approach that aims to control known offenders, for example, registries, background employment checks, longer prison sentences and various intervention programs. It is a tertiary prevention initiative that acts mostly in the individual sphere and, as such, presents certain inherent limitations in regards to preventing CSA from happening in the first place [107]. Indeed, although the public generally approves of so-called punitive legal practices, such as longer sentences, they are based on a misconception of sexual abusers as pedophiles, “guileful strangers” who prey on children in public places, when in actual fact the child sex offender population is more varied, includes individuals known to the victim and is comprised of juveniles in almost a third of cases [107].

The second most frequent approach, primary prevention, involves universal educational programs generally delivered in schools and aimed at potential victims. In the majority of cases, these universal programs also intervene in the individual preventive sphere and more infrequently in the family or societal sphere. Regarding children attending elementary school, meta-analyses by Zwi and colleagues [108], covering 15 studies, and by Davis and Gydicz [109], covering 27 studies, revealed that programs are effective at building children’s knowledge about sexual abuse and their preventive skills. The second of those two meta-analyses further demonstrated that programs are more effective if they are longer in duration (four sessions or more), if they repeat important concepts, if they provide children with multiple opportunities to actively practice the taught notions and skills, and if they are based on concrete concepts (what is forbidden) rather than abstract notions (rights or feelings). Some programs have proven effective for building knowledge and skills among children in an average socio-economic environment [110], but presented mitigated results in a multi-ethnic and underprivileged urban environment, indicating that the program may need to be adapted in order to optimize its effects with specific clientele [111]. As per adolescents or young adults attending high school or college, a meta-analysis of 69 studies involving close to 20,000 participants revealed that programs are effective for improving participants’ knowledge and attitudes [112]. However, changes in terms of behaviours or intentions to act were too low to be clinically significant. Also, factors related to the clientele, the facilitator, the setting and the format of the program have all been shown to impact the effectiveness of sexual violence prevention programs in college or university settings [113]. For some of the above programs, data are available to suggest that they are associated with a reduction of the incidence of child sexual assault [114] and sexual victimization in teenage romantic relationships [115]. However, too few studies are available to draw a firm conclusion as to the efficacy of prevention efforts, introduced since the 1970s, to reduce the true incidence of CSA observed by authorities in some countries, most notably the US [116-119].

The advantages of the universal approach are numerous: these programs can be offered at low cost, they are fairly easy to implement widely, and they allow to reach a maximum number of children while avoiding the stigmatization of a particular population. Yet, this approach has also been criticized since it places the responsibility of prevention in the hands of children. Consequently, this approach should not be considered as the only answer to a social problem as complex as CSA. A multi-factorial approach may indeed constitute a more promising solution to solve the problem of sexual abuse. A multi-factorial conceptualization of sexual assault suggests that only the development of global preventive approaches, targeting personal, family as well as societal norms that influence the risk of assault, may substantially reduce incidence and prevalence rates [119,120]. Those actions may take a variety of forms, such as awareness campaigns, efforts to provide the proper training to all persons who may work with children and adolescents, including sexual abuse and trauma themes in academic programs of future practitioners, or even the development of up to date and comprehensive kits to help the media provide information free of sexism, prejudices and sensationalism when reporting on sexual assault cases. In addition, parents’ participation is a fundamental element for a successful prevention initiative as this may increase the acquisition of preventive abilities in children [110], thus, future endeavors will need to tackle the challenges to foster a greater participation of parents. While most prevention initiatives have favoured a universal approach, targeting at-risk groups may also ensure optimal efficacy of prevention efforts. Integrating new technologies and using social medias (web site, applications for cell phones, online interactive games) may be particularly relevant for prevention efforts targeting teenagers. If such approaches were implemented and coordinated on a broad scale, they may have a greater impact on the number of sexual assault victims.

## Conclusion

The sexual abuse of children is a form of maltreatment that provokes reactions of indignation and incomprehensibility in all cultures. Yet, CSA is, unfortunately, a widespread problem that affects more than 1 out of 5 women and one out of 10 men worldwide. This alarming rate clearly calls for extensive and powerful policy and practice efforts. While the effects of CSA may not always be initially visible, survivors of CSA still carry the threat to their well-being. The traumatic experience of CSA is one major risk factor in the development of mental health problems affecting both the current and future well-being of victims. Considering that many victims continue to be undetected, the roots of these mental health problems may also be unrecognized. In an effort to provide effective services to all victims, we should prioritize the development of strategies to address the barriers to disclosure and reporting. Although the taboo of CSA might not be as prominent as a few decades ago when CSA was rarely spoken of, veiled issues may still prevent victims from reaching out to authorities to reveal the abuse they suffer. To effectively prevent CSA, global preventive approaches, targeting personal, family and societal conditions, need to be explored and validated so to protect the next generations of children and youth from sexual victimization.

## Competing interests

The authors declare that they have no competing interests.

## Authors’ contributions

The project was initiated by Prof. Dr. Collin-Vézina who wrote the sections on prevalence and mental health outcomes of CSA. Prof. Dr. Daigneault and Prof. Dr. Hébert led the writing on CSA prevention strategies. All authors read and approved the final manuscript.

## Author’s information

Prof. Dr. Delphine Collin-Vézina is the Tier II Canada Research Chair in Child Welfare. She is a clinical psychologist by profession and a researcher in the area of child sexual abuse. She is an Associate Professor at the McGill University School of Social Work (Canada). Her proposed research program aims at promoting societal recognition of sexual abuse, and at implementing and evaluating promising practices to help victims of abuse heal from their trauma.

Prof. Dr. Isabelle Daigneault is a clinical psychologist and an Associate Professor in the Department of Psychology at the Université de Montréal (Canada). She has a particular interest in the areas of resilience and mental health of young sexual assault victims, as well as in the processes influencing the life trajectories of young victims. Her projects also relate to the efficacy of treatments offered to victims and sexual assault prevention programs.

Prof. Dr. Martine Hébert has training in child development and child clinical psychology. She is Full Professor at the Department of Sexology at the Université du Québec à Montréal (Canada) and director of the Research Team on interpersonal trauma. Her research interests focus on the diversity of profiles in sexually abused victims and factors related to resilience trajectories. Current projects also center on the evaluation of prevention and intervention programs.
